# The Trickle-Down Effect of Responsible Leadership on Employees’ Pro-Environmental Behaviors: Evidence from the Hotel Industry in China

**DOI:** 10.3390/ijerph182111677

**Published:** 2021-11-07

**Authors:** Hong Tian, Danni Suo

**Affiliations:** Business School, Jilin University, Changchun 130012, China; tianhong2919@163.com

**Keywords:** responsible leadership, trickle-down effect, employee pro-environmental behavior, hotel industry, the perceived role of ethics and social responsibility

## Abstract

Based on the trickle-down effect model, social learning theory and trait activation theory, this study explores the mechanisms of multi-level responsible leadership on employees’ pro-environmental behaviors in the hotel industry in China. The results show that responsible leadership positively influences employees’ pro-environmental behaviors; mid-level responsible leadership significantly mediates the positive relationship between high-level responsible leadership and employees’ pro-environmental behaviors; and the perceived role of ethics and social responsibility positively moderates the relationship between responsible leadership and employees’ pro-environmental behaviors. The results of this study provide empirical support for further analysis of the “black box” of responsible leadership on employees’ pro-environmental behaviors, fill the gap of the trickle-down model in leadership, and provide new directions for sustainable value creation in hospitality industry organizations.

## 1. Introduction

Following the global resumption of work in the post-epidemic period, more responsible and environment-benign corporate performance demands attention for all global enterprises, especially in the hospitality industry, which is a labor-intensive industry and one that requires considerations for the ongoing and perspective economy, society, and environment [1,2,3]. The question of how to accelerate the transition to a sustainable future for the hospitality industry has become critical in achieving effective environmental management [4,5]. In addressing this issue, researchers have investigated, from various perspectives, including applying competitive synergies, CSR as tools, environmental policies and practices, and sustainable supply chain management in hospitality enterprises [4,6,7,8]. However, rare studies focus on the indispensable role of employees and their contribution to the sustainability and effective environmental management in the hospitality field [9]. Thus, this study selected employee pro-environmental behavior, which encourages employees to promote the spillover effects of extra-role behaviors based on in-role behaviors to achieve organizational performance goals, reflecting the consistency between employees and organizational environmental responsibility values, social responsibility values and their conceptual goals of sustainable development [10] as a focus of research to fill the gap. When organizations and employees are committed to the environment at the same time, environmental sustainability and organizational sustainability can simultaneously be achieved, and organizations can achieve economic, social, and ecological benefits [11].

Existing studies have conducted research on employee pro-environmental behavior from perspectives of work behaviors (such as job performance, creativity, or citizenship behaviors), driving forces, moral characteristics, and person–environment relations [10,12,13,14,15], while in organizations, leaders are more prominent objects of employee observation. Consequently, some scholars have attempted to probe the facilitators of employee pro-environmental behavior from the viewpoint of leadership research [16,17,18]. However, most of the existing studies on leadership and employee pro-environmental behavior have focused on leadership styles with a single core of business owners, such as ethical leadership [19] and transformational leadership [20], and studies have shown that these types of leaders can promote employees’ pro-environmental behaviors in the corresponding settings. However, leadership research with a single group as the core has limitations in addressing employees’ pro-environmental behaviors, lacks sufficient fit with corporate social responsibility and ethical values, and fails to respond positively to stakeholders’ needs. Based on this research, the paper is designed from a multi-level perspective of integrated corporate strategy and selects responsible leadership, which is concerned with the desires of stakeholders who are internal and external of the organization, create value in an environmentally sustainable manner, and possess the attributes of a leader trait that fully implements corporate social responsibility for organizational citizenship behavior with responsible values as the core idea [21]. Responsible leadership is a powerful complement to existing leadership trait research frameworks and leadership theories, with its ability to respond to individual, organizational, and systemic levels of scandalous, novel society, ethical, and environmental challenges that arise from environmental issues [22].

When associates feel backed by their leaders, they will be far more motivated to take environmental initiatives to help the company achieve its sustainability goals and create long-term competitive advantage. With sustainability as an organizational goal, members at different levels of the organization can contribute to environmental protection in different directions [23]. Senior leaders make corporate green purchasing decisions, mid-level leaders are able to propose solutions that promote environmental orientation in the market, and grassroots employees can demonstrate environmental attitudes and behaviors at work [24]. Following social learning theory, employees usually learn and imitate the behavior of their superiors. Thus, in the worksite, employees will pattern their own behavioral traits by ascribing the behavior of their superiors [25]. By this logic, lower-level managers can also evolve their own leadership traits by learning and ascribing the behaviors of higher-level managers [26]. Therefore, this paper infers those top-level responsible leaders are likely to influence the behavior of mid-level responsible leaders. In addition, given the role modeling effect of mid-level leaders on employees and the existing research on the construction of trickle-down effects of similar leadership behaviors, such as ethical leadership [27], this paper proposes a trickle-down model of responsible leadership in which the top levels of responsible leadership have an influential role in employees’ pro-environmental behavior that is indirectly generated through mid-level responsible leadership.

In addition, not all employees learn and imitate the behaviors of their superiors, and the leader’s role model effect is influenced by many environmental and individual factors, such as the traits of the observer [28]. Employees’ perceptions at the psychological level determine to a certain extent the effectiveness of leadership. Trait activation theory states that an individual’s conduct is the consequence of the mutual interplay of personal traits and situational determinants [29]. As an essential contextual driver of employees’ pro-environmental behaviors, the perceived role of ethics and social responsibility (PRESOR) affects the strength of employees’ perceptions of social and environmental responsibility issues in the workplace [30]; this then affects employees’ pro-environmental behaviors and ultimately contributes to the enhancement and prosperity of environmental sustainability. Therefore, this paper incorporates trait activation theory to explore the boundary conditions for the effectiveness of the role of responsible leadership on employees’ pro-environmental behavior.

This study is an extension of the line of research on leadership and pro-environmental behaviors in the worksite in three ways. First, the trickle-down effect is selected in this paper as a research framework and methodological innovation, it introduces a process perspective based on the trickle-down model to probe the mechanism of responsible leadership on employee pro-environmental behavior holistically and dynamically, and to explore the multi-level dynamic diffusion effect of the top and the mid responsible leadership on employee pro-environmental behavior in the path, which makes up for the single-level, static study of managers’ behavior path between the leader–employee level in previous studies. Second, this paper bridges a gap in the hospitality field concerning the perspective of employee behavior and empirically tests the necessity for follow-up research. Third, this paper selects PRESOR as the moderating factor, which is conducive to deepening the contextualization study of responsible leadership, and extends the boundary of trickling effect conditions and advances our comprehension of the trickling process of leader-to-follower conduct. In response to the call for a multi-level analysis of the antecedents and consequences of responsible leadership [31], we pioneered the study of the effect of senior leaders’ responsible leadership on employees’ pro-environmental behaviors through low-level leaders’ responsible leadership, additionally validating the existence of low-level leaders’ responsible leadership but also enriching the multi-level study of responsible leadership.

## 2. Theory and Hypotheses

### 2.1. Responsible Leadership and Employee Pro-Environmental Behavior

Responsible leadership is a blend of social responsibility, morality, and leadership from a stakeholder perspective [32,33]. It involves a leadership style in which the leader demonstrates responsible behavior that reconciles the needs of all parties in order to achieve sustainable organizational value co-creation and social change [34]. Responsible leadership emphasizes effectiveness, ethicality, sustainability, legitimacy, and accountability. It breaks the deadlock of the traditional static binary relationship model study of the previous study of leader–subordinate, introduces a dynamic multi-interaction model study with stakeholders as the object of consideration, and incorporates the consideration of ethical constraints into decision-making, which is an effective complement to the previous studies of leadership effectiveness. In contrast to the numerous ethical leadership types previously studied, responsible leadership centers on sustainable value generation through constructive social impact [35] and is an effective bridge across firm-level social responsibility and individual-level leadership responsibility [36]. At this stage, the research concerning employee pro-environmental behavior has shown an initial trend, but unlike general pro-environmental behavior, employee pro-environmental behavior in the worksite is contextualized by the organization, and the resulting environmental behavior will have a beneficial interaction with organizational decisions, which will advance the attainment of an organization’s greening policy and the achievement of environmental sustainability [37].

According to social learning theory [25], individuals learn and develop appropriate behaviors by imitating the conduct of others. In the workplace, employees imitate their superiors as role models because of the authority and charisma they possess. Unlike traditional leaders who seek to maximize profits and benefits, responsible leaders respond to and coordinate the actions of stakeholders inside and outside the organization by building sustainable trusting relationships with them to achieve the win–win goal, a shared business vision, and sustainable value creation [38]. This responsible behavior possessed by the leader and a purpose to motivating stakeholders, set a model for employees, since employees may imitate leaders in fulfilling their social responsibilities and reduce irresponsible behaviors in the worksite. According to the definition of employee pro-environmental behavior, it refers to all voluntary behaviors of individuals to conserve the environment or better organizational practices or processes in environmental management [39], reflecting the alignment of employees with the organization’s socially responsible as well as environmentally responsible values, beliefs, and goals, and having an overall positive effect on organizational goals [10]. Thus, in directions led by responsible leaders, employees, through imitative learning as well as following their leaders, will realize and understand the importance of the three attribute characteristics of pro-social, spontaneous, and extra-role behaviors of employees’ pro-environmental behaviors [40], and thus increase their willingness to attend to employees’ pro-environmental behaviors.

Responsible leadership is viewed as a shifting procedure of transferring social relations in the organization, identifying subordinate employees as the primary stakeholders in the organization. When employees are the primary stakeholders in the organization, employees associate themselves with their leaders and the organization to realize their value and gain a sense of identity [41]. Responsible leaders serve as role models and enhance the emotional involvement of subordinates, which in turn enhances their sense of identification and attachment to the organization. Employees who are overseen by responsible leaders are at a greater potential to connect their own values with those of the organization [28]. Responsible leaders actively assume social responsibility and encourage employees to work towards a vision of environmental sustainability based on the triple bottom line principle. The values of responsible leaders are congruent with the values of the organization and serve as role models for subordinates to learn how to lead the organization. According to the trickle-down effect model, subordinate employees learn from higher-level leaders. Thus, when influenced by responsible upper-level leaders, subordinate employees’ sense of responsibility is stimulated and reinforced, resulting in the adoption of the upper-level leader’s responsible attitude toward stakeholders and the performance of similar behaviors. Although lower-level employees and higher-level leaders are faced with different work tasks, both following the same ethical guidelines. Responsible leaders are attending to the needs of the natural environment, following a sound ethical logic and seek sustainable value creation through a positive social role, are able to fulfill environmental responsibilities, and develop and enhance processes related to environmental management in their organizations. Therefore, this paper argues that responsible leaders can actively promote pro-environmental behavior among subordinates in the organization and provide a balance between near-term economic returns and late-term environmental gains. Specifically, we hypothesized the following:

**Hypothesis** **1** **(H1).** *Responsible leadership is positively related to employees’ pro-environmental behaviors.*

### 2.2. The Mediating Role of Mid-Level Responsible Leadership

Although previous research has shown that all dimensions of the organization show favorable intentions to promote environmental protection, supervisors are more possible for engaging in environmentally friendly conducts than subordinates, who usually need more attention and support from their superiors to perform in a more environmentally friendly manner [23]. Based on social learning theory, it is believed that subordinates usually learn and model the behavior of their superiors. When organizations emphasize environmental concerns and help, employees are more likely to be involved in environmentally responsible behavior to help the organization become sustainable [42].

As mentioned earlier, subordinates are able to mold and formulate their own behavioral traits by learning and replicating the acts of their superiors [25]. In this logic, lower-level leaders can also learn appropriate behaviors from senior leaders, i.e., senior leaders actively take social responsibility and make efforts to achieve sustainable creation goals, and lower-level leaders recognize and model this leadership model, learn, and imitate it, and develop their own behavioral styles [26]. According to social learning theory [25], lower-level leaders are activated and strengthened by the influence of responsible leadership from senior leaders, which leads to the adoption of responsible stakeholder attitudes and similar socially responsible behaviors from senior leaders. Although lower-level leaders and senior leaders face different stakeholders, the guidelines for coexistence with stakeholders are the same. Thus, according to the practices of top responsible leaders, lower-level leaders respond positively to the demands of their subordinate employees and effectively weigh their problems in the process of working together and collaborating with them. As a corollary, what employees learn from leaders varies slightly, given that leaders at different levels have different capabilities. Employees tend to learn the behavioral norms needed for their jobs from lower-level leaders, while they learn the organization’s policy guidelines and establish values from higher-level leaders. In addition, the intensity with which employees learn from leaders at different levels also varies. Lower-level leaders have a stronger influence on employee behavior than top-level leaders because they have a closer relationship with employees at work [43].

In line with Hypothesis 1, this paper further infers that the interpretation of responsible leadership upon employee pro-environmental behavior is realized through a trickle-down model, i.e., responsible leadership is transmitted and diffused through the organization from top to bottom in the vertical management hierarchy to influence employee pro-environmental behavior [44]. On one side, top responsible leaders serve as the setters of institutional policy guidelines, dominate the organization’s culture of responsibility [34], and influence employees’ pro-environmental behaviors in the worksite. On thenother side, mid-level leaders are inspired by the responsible attitudes and behaviors of senior leaders and follow and spread the influence of the responsible culture of senior leaders, thus motivating employees’ pro-environmental behaviors. Therefore, this paper considers the relationship between mide-level responsible leadership mediating top-level responsible leadership and employee pro-environmental behavior. These arguments suggest the following hypothesis:

**Hypothesis** **2** **(H2).** *Mid-level responsible leadership has a mediating influence between top-level responsible leadership and employees’ pro-environmental behaviors.*

### 2.3. Moderating Role of the Perceived Role of Ethics and Social Responsibility (PRESOR)

The perceived role of ethics and social responsibility refers to the significance of moral and social responsibility perceived by individuals in the decision-making process, which as a situational factor affects the strength of individual’s perceived social responsibility issues in the workplace [30]. When individuals are faced with work decisions, a strong sense of ethical and socially responsible roles can motivate them to engage in responsible behaviors that are more ethical and moral and beneficial to organizational performance and social welfare. Empirical studies have shown that employees in environments with strong ethical and socially responsible role perceptions have significantly differentiated their own mental states (attitudes, cognitions, and feelings) and behaviors compared to weak ethical and socially responsible role perception environments [45]. According to trait activation theory, an individual’s acts are the result of the interplay between individual traits (personality, values) and situational factors (leader support, ethical and social responsibility perceptions). Contextual factors stimulate employee traits and effectively predict the corresponding behavior. Contexts with high ethical and socially responsible roles can effectively motivate employees to be environmentally conscious, engage in pro-environmental behaviors, and promote alignment with the organization’s ethical values, sense of responsibility, and environmental philosophy. Therefore, employees with responsible leaders are more likely to use perceived leaders’ environmentally responsible values as goal signals and integrate them with their own values, pursue long-term goals for organizational environmental management, and establish strong social-emotional connections with organizational environmental sustainability. Thus, we hypothesize the following:

**Hypothesis** **3** **(H3).** *The perceived role of ethics and social responsibility plays a moderating role between responsible leadership and employee pro-environmental behavior.*

**Hypothesis** **4** **(H4).** *When PRESOR is strong, ethical and social responsibility plays a positive moderating role between top-level responsible leaders and employees’ pro-environmental behaviors.*

**Hypothesis** **5** **(H5).** *High levels of PRESOR are contingent on the indirect effect of top-level responsible leadership on employees’ pro-environmental behavior through mid-level responsible leadership.*

**Figure 1:**
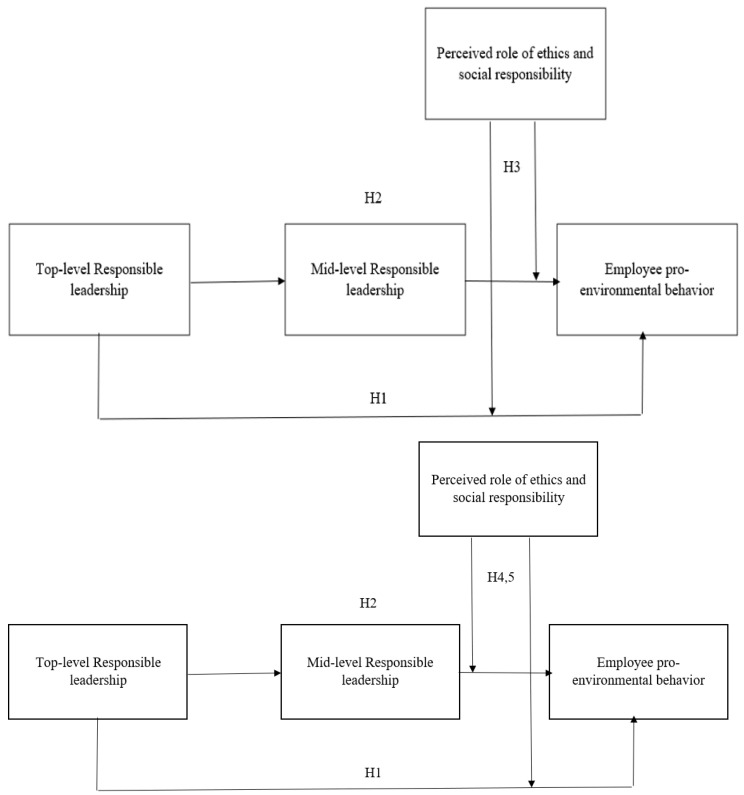
Theoretical model of the trickle-down effect of responsible leadership on employee pro-environmental behavior. H1: direct effect. H2: indirect effect. H4 and H5: contingent effects (moderated mediation).

The theoretical model diagram of this study is shown in Figure 1.

## 3. Methods

### 3.1. Design, Sample, and Participants

The hospitality industry is one of the main forces of China’s economy and advances the globalization among the nations’ trade. Although the short-term growth rate of economic indicators is relatively impressive, the gradually emerging problems of environmental degradation and imbalance of environmental indicators have become substantial blockages to the progress of sustainable development. Within the hospitality industry, specifically the hotel sector, efforts should be emphasized to mitigate the environmental impact due to its 24-h service, excessive energy consumption, and wastage of other resources. Therefore, we used the hotel industry of the hospitality field as the survey sample and selects managers and their subordinates from hotels with star-level in the Jiangsu and Zhejiang (regions with developed hospitality industries) of China.

A convenience sampling method was used and it was structured in an effort to allow all respondents to participate on a voluntary basis after reading the purpose of the study. We coded the questionnaires before distribution, and distributed them on site by anonymously. Once the questionnaires were completed, they were sealed in an envelope and delivered directly to the mailbox. To reduce potential common method bias, this study used a three-stage data collection approach with two months between each study and a corresponding code designed in each study to match the questionnaire three time-points. At time-point one, the socio-demographic information was collected. At time-point two, PRESOR and employee pro-environmental behavior were gathered. At time-point three, items to measure responsible leadership were obtained. About 750 participants were randomly selected from star-level hotels in China, including a star-level over 2 stars. Ultimately, A sum of 561 people enrolled in the study by filling out a valid questionnaire after three matches. The overall response rate was 74.80 %, a total of 56.10 percent were male, with an average age of 38.30 years (SD = 0.49, ranged from 20 to 53 years) and an average organizational tenure of 11.08 years (SD = 1.22). Finally, A university degree was held by 78.40 % of those polled.

### 3.2. Measures

The measurement scales selected for this paper were all validated scales with balanced reliability and validity and were translated-back-translated [46] to ensure consistency in meaning of items. They were also verified to ensure the accuracy of the question statements and the high fit between the measured variables. Items were scaled on Likert’s 5-point scale, with 1 standing for strong disagreement and 5 standing for strong agreement.

Responsible leadership was measured through 5-item scale selected from Voegtlin [47]. The coefficients α of reliability were 0.90 (M = 3.52; SD = 0.99) and 0.95 (M = 3.36; SD = 0.98) for the employees’ perceived high-level and mid-level responsible leadership respectively.

Employee pro-environmental behavior stems from a 6-item scale designed by Robertson & Barling [17]. The Cronbach’s α value for this scale was 0.87 (M = 4.10; SD = 0.70).

PRESOR was rated in a 5-item scale that proposed Etheredge [48]. The Cronbach’s α value for this scale was 0.92 (M = 3.95; SD = 0.88). In order to reduce the intervention of irrelevant variables on the research variables in this paper and to effectively ensure the explanatory validity of the empirical test results on the theoretical framework of this paper, gender, age, education, and length of tenure were formulated as control variables.

## 4. Analyses and Results

This study consists of performing conditional process analysis (CPA). According to Hayes [49], CPA is recommended when the “research goal is to understand and describe the conditional nature of the mechanism or mechanisms by which a variable transmits its effect on another and testing hypotheses about such contingent effects” [49] (p. 395). In this study, CPA was performed in the form of moderated mediation by which the strength of the indirect effect of top-level responsible leadership (independent variable-IV) on pro-environmental behavior (dependent variable) through mid-level responsible leadership (mediator) was contingent on the levels of the perceived role of ethics and social responsibility (moderator). This was performed through the Macro process Model 15 [49] (p. 592). For decision-making, the rule of thumb is that the value of both direct and indirect effects should be different from 0 (with n = 5000 bootstrap resamples). This means that the confidence intervals (CIs) should not contain 0. Finally, the index of moderated mediation was used as an inferential test [50]. The index is significant if the CIs do not include 0.

### 4.1. Common Method Bias

Common method variance (CMV) was checked by performing the market technique [51]. This technique consists of loading all focal indicators of the investigated variables onto one theoretically unrelated maker variable. Williams et al. [52] defined a marker as a variable capturing or tapping into one or more of the sources of bias that can occur in the measurement context for given substantive variables being examined (p. 507). Table 1 reports that all substantive indicators loaded significantly on their hypothesized factor (*p* < 0.001). Also, following the recommendation by Lindell and Whitney [53], Table 1 shows that the marker variable has a correlation close to 0 with at least one substantive variable (i.e., top level responsible leadership). The text of the scale items is showed in the Appendix A at the end of this paper.

Finally, Table 2 provides the baseline comparison. Findings indicate that the measurement model offered a better fit than the measurement model with the marker variable. First, Chi-square difference test [54] was significant (∆χ2 = 70, *p* < 0.001). Second, following the recommendation by Hu and Bentler [55], the measurement is more parsimonious since it has the lowest AIC (AIC = 699.9). In sum, based on all these different cues, we concluded that in this research, CMV was not a serious threat.

### 4.2. Confirmatory Factor Analysis

To be enabled to validate the effects of the main variables “high-level responsible leadership”, “mid-level responsible leadership”, “employee pro-environmental behavior”, and “PRESOR”, a four-factor model with structural equations was used. The measurement model fits the data well, χ2(144) = 607.9, *p* < 0.001, CFI = 0.95, NNFI = 0.95, RMSEA = 0.07.

Table 1 reports the main psychological properties. The dimensionality of each variable was all satisfactory, since they are all above the recommended cut-off 0.50 [56] and 0.70 [57] for the average variance extracted (AVE), and the Jöreskog’s rho (ρ), respectively. In this research, AVEs ranged from 0.55 to 0.71, and ρs from 0.85 to 0.92.

Additionally, to guarantee the variables’ distinctiveness, the discriminant validity was assessed. Following Fornell and Larcker [57], discriminant validity between two variables is evidenced when their shared variance is lower than the average of their respective AVE. Shared variances appear in parentheses in Table 3 and consist of their squared correlation. For example, as expected, the highest correlation is shown between top-level responsible leadership and mid-level responsible leadership (since they derived from the same statement); their shared variance is about 0.59 (0.77 × 0.77); their AVE mean is 0.69. On this basis, their discrimination is evidenced. The results in Table 3 show that the basic criteria are met for all pairings of variables of the research.

In sum, convergent validity, dimensionality, and distinctiveness of the data are evidenced.

### 4.3. Descriptive Statistics

The output of the descriptive statistical analysis and correlation coefficient matrix of the variables of concern in this research are included below (see Table 3). According to the correlation coefficient matrix, top-level responsible leadership, mid-level responsible leadership and employee pro-environmental behavior were significantly positively correlated (r = 0.48, *p* < 0.01; r = 0.49, *p* < 0.01). The research hypothesis of this paper was initially verified. As showed in the Table 2, the discriminant validity test holds if indeed the square root of a variable’s average variance extracted (AVE) is larger than the correlation coefficient of this variable as well as other variables.

### 4.4. Hypotheses Testing

Table 4 reports findings regarding both H1 and H2. Consistent with H1, responsible leadership has a positive and significant effect on employees’ pro-environmental behaviors (b = 0.18, SE = 0.02, t = 4.51, *p* < 0.001). Consistent with H2, the indirect effect of top-level responsible leadership on employee pro-environmental behavior through mid-level responsible leadership was significant since the 95% confident intervals do not straddle 0 (0.16, SE = 0.03, 95%CI = 10, 21).

Table 5 reports findings concerning H4 and H5. Before performing both hypotheses, the product terms for H4 (top-level responsible leadership x PRESOR) and for H5 (mid-level responsible leadership) were mean-centered to avoid multicollinearity [58]. H4 anticipated that the direct effect of top-level responsible leadership on employee pro-environmental behavior would be stronger at high versus low levels of PRESOR. Findings indicated that the effect of top-level responsible leadership on pro-environmental behavior is moderated at low levels of PRESOR because 95% CI does not contain 0 (0.24, 0.41) and no such moderating was found at high levels of it since 95% CI contains 0 (−0.08, 0.07).

H5 anticipated that the indirect effect of top-level responsible leadership on employee pro-environmental behavior through mid-level responsible leadership would be stronger at high versus low levels of PRESOR. Consistent with the expectation, findings reported that the conditional indirect effects of top-level leadership on pro-environmental behavior is moderated at high levels of PRESOR since 95% CI does not straddle 0 (0.05, 0.20), whereas no such moderating effect was found at low levels of it because 95% CI includes 0 (−0.10, 0.12). In addition, the index of moderated mediation that serves as an inferential test [59] demonstrates the significance of moderated mediation because it is different from 0 (Index = 0.06, BootSE = 0.04, 95% CI = 0.005, 0.137).

In summary, results for H4 (although at low rather high levels) and H5 support the expectation of H3 regarding that the effect of responsible leadership on employee pro-environmental behavior is moderated by the perceived role of ethics and social responsibility.

## 5. Discussion

### 5.1. Research Findings

Through incorporating social learning theory, this research intended to construct a trickle-down model of responsible leadership on employee pro-environmental behavior in a Chinese organization context and explored the boundary effects of PRESOR in the intrinsic impression mechanism of responsible leadership upon employee pro-environmental behavior based on trait activation theory and obtains the following findings. The far more important result is that the transmission of impression of top responsible leadership upon employee pro-environmental behavior through mid-level responsible leadership is strengthened when the PRESOR is low, while the transmission of effect of mid-level responsible leadership on employee pro-environmental behavior is reinforced when the PRESOR is high.

This study supplements the environmental literature, as the findings provide a broader and diverse understanding of how the multi-level transmission mechanism of responsible leadership effectiveness from a process viewpoint and adds the consideration of multi-level transmission effects within the organization to the traditional, static, and single-level study of the leader–employee role model.

Regarding the advancement of PRESOR, there is one critical finding worth highlighting relates to employee pro-environmental behavior when considering differ level of responsible leadership. In this respect, Table 4 provides an empirical representation, which represents the main outcomes regarding hypotheses H4 and H5. However, although the results for the H4 is significant, it showed the results at low rather than high levels.

Several attempts have been made to explain the counterintuitive data results. First, this paper is based on the hotel industry as the subject of the data sample selection for the empirical test, and the path to enhance employee pro-environmental behavior studied in this paper also has more theoretical and practical application to the hotel industry (an industry that is more likely to pollute the environment). The hotel industry itself has a certain amount of unavoidable negative impact on the environment; therefore, for the PRESOR scale consisting of multi-perspective items, the observed sample of the hotel industry considers ecology as a lower impact social responsibility level in PRESOR. This inference also echoes [60] in establishing the PRESOR scale, for managers to place ecological factors in social responsibility as the least important indicator. In addition, managers’ decision-making processes are not clearly observable when allocating resources in the organization, especially limited and scarce resources, or when faced with the overriding needs and intense competition from stakeholders outside the organization. As a result, top managers are highly likely to allocate resources to other areas of the organization and reduce the proportion of ecology in social responsibility. According to social learning theory, when employees observe top managers’ consciousness of the low role of ecology in social responsibility, employees will correspondingly adjust the implementation of pro-environmental behaviors.

Second, researchers have previously examined the differences in ethical perceptions between different hierarchical positions in organizations [61,62]. This paper argues that the differing ethical perceptions of top and mid-level managers are caused by the differences in the critical stakeholders, the focus of work goals, and the sources of stress. Regarding the stakeholder perspective, top managers are responsible for developing corporate strategy, the high visibility of their organizational position puts them under undue pressure, and they need to encounter pressures from shareholders, the media, the public, government, competitors, and the environment; in particular, they need to balance these pressures with the different focuses imposed on executives. For more details, senior executives might prioritize the most optimal resources that can achieve short-term results, such as short-term financial growth and reputation building for marketing and charitable contributions when allocating corporate resources, because stockholders need senior executives to focus on stable growth in financial profitability while government, society, and the media expect executives to focus on maintaining corporate reputation. The environment is a long-term investment in the organization, and its sustainable growth needs to be observed through long-term investment, so top managers may neglect to prioritize the environment in their strategy. In contrast, the main stakeholders faced by mid-level managers are employees and superiors. Managers need to ensure that their own behaviors and those of their subordinates are regulated and that environmental policy assessment targets are met, without having to consider too much the planning of external corporate image-building activities, as such. Thus, when faced with high PRESOR, mid-level managers’ perceptions of social responsibility and ethics will emphasize the environmental aspects and raise their concern for the environment, thus regulating their own behaviors and those of their subordinates, which in turn will further promote pro-environmental behaviors of employees.

Third, the distinction in the ratio of men to women and age distribution among top and middle managers also has some influence on ethical perceptions. As opposed to males, females are more sensitive to moral issues and thus identify more with ethical concerns [63,64]. In the same situation, the focus of ethical decision-making differs between male and female managers, e.g., women will be more inclined to ethical perceptions of interpersonal relationships and empathy, while men will focus more on ethical justice such as organizational rules and individual rights [65,66]. According to the statistics posted by the Peterson Institute for International Economics in November 2020, the percentage of female officers is considerably underrepresented as compared to the proportion of male officers in China. This result is not specific to China, but even in Europe, which has a high proportion of female executives in global terms, the average is only 22.60%. While Asian countries are influenced by the traditional values, the overall average figure is low. Thus, the predominance of male executives causes the overall level of ethical perceptions to be lower than that of mid-level managers, which leads to insensitivity to environmental ethical issues in business operations, further weakening the positive promotion of pro-environmental behavior among employees. Additionally, as individuals get older, their ethical perceptions and attitudes become correspondingly conservative, and they are less liberal in observing and judging the potential ethical situations surrounding them [67]. This study deepens the contextualization of responsible leadership and introduces ethical and social responsibility variables (PRESOR) to explore the boundary effects of the psychological response mechanism of responsible leadership to employees’ pro-environmental behavior, which provides a new paradigm for theoretical research on environmental psychology and corporate ethics in corporate sustainable development in the hospitality industry.

### 5.2. Practical Implications and Prospects

The practical implications of this study are summarized as follows: First, corporate leaders should focus on their own exemplary role and internalize the rigid requirement for subordinates to focus on environmental sustainability enhancement from the leader’s intrinsic attitudes, attributes, and behaviors to the employees’ esteemed standards, which translates into flexible interventions in the behavior of the employee group. Specifically, responsible leadership with sustainable value creation as the core goal and business vision, and ecology as a consideration is a better way to advocate pro-environmental behavior in hotels. Furthermore, the different perceptions of ethical and social responsibility faced by top and mid-level managers in this paper result in different degrees of promotion of pro-environmental behavior among employees, suggesting that hotels should take diversity into account in the selection of managerial staff, both at the top and mid-level managerial levels.

Second, leadership at different levels of the hotel should continue to cultivate and develop the attributes and behaviors required for responsible leadership to employees at lower levels of the hotel through continuous improvement of their own responsibility values and behaviors, so that effective leadership starts from within the organization and encourages employees to shift from passive compliance with organizational environmental regulations to active environmental responsibility, and from economic growth to recognition of ecologically sustainable development models, in order to achieve a mutual win–win situation in the sense of economic, environmental, and social performance.

Third, corporate sustainability management is a long-term environmental and social responsibility strategy that demands undertaking at all levels of the organization, thus, enterprises should ensure that their employees understand the commitment of the company in improving environmental and social achievements. Especially for the hospitality industry, which is a labor-intensive industry, sometimes unilateral communication of the company’s policy and determination in environmental management through media, training programs, annual reports, and so forth fails to make the staff fully understand the core ideas of the organization and may even have some hesitant attitude towards the business practices. Therefore, effective environmental management should focus more on caring for the thoughts and attitudes of employees in the organization, finding ways to communicate with them, explaining their worries about environmental management, and imparting environmental knowledge and setting up environmental philosophy, so as to cultivate employees’ commitment in environmental improvement and achieving corporate greening.

### 5.3. Limitation and Future Research

Despite some interesting findings in this study, there remain limitations that need to be further refined in future studies.

First, although a large sample size was selected for this paper, the samples are concentrated in the hotel industry, and this study does not account for industry effects, which would allow for intra-industry comparisons. Specifically, the data were mostly selected from star-level hotels, and the effectiveness of the theoretical model in other scales and industries is still debatable, thus subsequent studies can further focus on other industries to increase the external validity of the study. Moreover, since this study examined hotels situated in China, it remains to be deter-mined how generalizable our findings are to other industries and across cultures. More specifically, it is unclear whether our data are biased by the unique characteristics of the hospitality industry, given that it is more reliant on resources than other industries. Therefore, prospective research should attempt to replicate our findings in other organizations’ settings and cultures.

Second, regarding the selection of the sample of top management, this paper does not further refine them, such as separating them by functions. For the critical factor of PRESOR, the impact of top managers on ethical perceptions and pro-environmental behavior of employees may differ depending on the authority they are responsible for. Furthermore, the bipartite ethical decision-making process, consisting of teleological evaluation and deontological evaluation, may have an impression upon perceptions of social responsibility and ethics due to individual characteristics of administrators, especially in achieving long-term organizational performance, and more explicitly, teleological evaluations would judge actions based on the results and impacts they generated, while deontological evaluations would be more norm-based in their judgment and assessment.

Third, limitations from the employee pro-environmental behavior are addressed. Although the scale selected for this study is widely used by scholars in examining pro-environmental behaviors in the workplace, the items in the scale refer to behaviors that many employees may use beyond the workplace; therefore, it is not critically possible to determine whether the conduct is established at work because of personal habits or cultivated by the organization [68]. Therefore, more workplace confined pro-environmental behaviors to measure should be probed in future studies.

## 6. Conclusions

The hospitality industry, the mainstay of world productivity, is also a labor-intensive industry that contributes to the employment of over 10% of the world’s workforce [69]. This industry, while contributing to the global economy, value and the meaning, also has inevitable repercussions on the environment, especially in the hotel industry. The pattern of extended hours of operation, excessive use of resources and reliance on them all cause detriments to the environment and resources. Relatively speaking, in light of the hospitality industry’s reach and exposure, a heightened awareness among hospitality stakeholders to become more responsible stewards of a sustainable future for humanity is critically needed. Questions of how to accelerate the transition to a sustainable future for the hospitality industry and build overall staff integrated effective environmental management are not meant to be solved in this paper. This research intended to construct a trickle-down model of responsible leadership on employee pro-environmental behavior in a Chinese hospitality organization context and explored the boundary effects of PRESOR in the intrinsic impression mechanism of responsible leadership upon employee pro-environmental behavior based on trait activation theory and social learning theory. Findings of the article theoretically and statistically validate the hierarchical cascading effect of responsible leadership to employee pro-environmental behavior across organizations and the differential strength of leadership across hierarchical levels. The present paper verified that responsible leadership has a positive and significant effect on employees’ pro-environmental behaviors. Mid-level responsible leadership has a mediating influence between top-level responsible leadership and employees’ pro-environmental behaviors. Moreover, lower-level leaders have a stronger influence on employee behavior than top-level leaders, which echoes the cascading effect. The perceived role of ethics and social responsibility plays a moderating role between responsible leadership and employee pro-environmental behavior. When PRESOR is strong, ethical, and social responsibility plays a positive moderating role between top-level responsible leaders and employees’ pro-environmental behavior. In addition, high levels of PRESOR are contingent on the indirect effect of top-level responsible leadership on employees’ pro-environmental behavior through mid-level responsible leadership. This study is an extension of the line of research on leadership and pro-environmental behaviors in the worksite. It not only selects the trickle-down effect as a research framework and methodological innovation, but bridges a gap in the hospitality field concerning the perspective of employee behavior and empirically tests the necessity for follow-up research. Effective environmental management should focus more on caring for the thoughts and attitudes of employees in the organization, finding ways to communicate with them, and imparting environmental knowledge and setting up environmental philosophy, so as to cultivate employees’ commitment in environmental improvement and achieving corporate greening. In closing, it serves to consider that organizational sustainability concerns and the implementation of ecological initiatives in the hospitality industry depend considerably on the commitment and practices of employees; leveraging this commitment is more likely when responsible leadership is engaged.

## Figures and Tables

**Table 1:** Standardized parameter estimates for measurement model with and without marker.

	Measurement Model					Measurement Model with a Marker Variable
			Responsible Leadership			
	Employees’ PEB	PRESOR	TOP	MID	MKR	
EPEB 1	0.773 ***					0.645 ***
EPEB 2	0.796 ***					0.646 ***
EPEB 3	0.588 ***					0.582 ***
EPEB 4	0.722 ***					0.590 ***
EPEB 5	0.826 ***					0.679 ***
EPEB 6 (removed)	0.473 ***					0.504 ***
PRESOR 1		0.616 ***				0.538 ***
PRESOR 2		0.971 ***				0.573 ***
PRESOR 3		0.945 ***				0.568 ***
PRESOR 4		0.821 ***				0.533 ***
PROSOR 5		0.696 ***				0.554 ***
TOP 1			0.789 ***			0.637 ***
TOP 2			0.786 ***			0.619 ***
TOP 3			0.865 ***			0.717 ***
TOP 4			0.847 ***			0.708 ***
MID 1				0.835 ***		0.539 ***
MID 2				0.897 ***		0.723 ***
MID 3				0.881 ***		0.704 ***
MID 4				0.852 ***		0.676 ***
MID 5				0.786 ***		0.612 ***
MKR 1					0.912 ***	-
MKR 2					0.926 ***	-
MKR 3					0.870 ***	-
MKR 4					0.904 ***	-
Cronbach alpha	0.87	0.92	0.90	0.95		
Joreskog Rhô	0.85	0.90	0.88	0.92	-	
Average variance extracted	0.55	0.66	0.67	0.71	-	
Correlation with marker	−0.167	−0.122	0.061	0.109	-	

*** *p* < 0.001, a variance for measurement model before the inclusion of marker; c variance for measurement model after the inclusion of marker.

**Table 2:** Results of Model Comparisons (N = 561).

Models	χ2	df	χ2/df	CFI	NNFI	RMSEA	AIC
Null model	12,858.4 ***	253	50.8	-	-	-	-
Measurement model with marker variable	677.9 ***	217	3.1	0.96	0.95	0.06	795.9
Four-factor model (measurement model)	607.9 ***	144	4.2	0.95	0.95	0.07	699.9
Three-factor model (top and mid together)	1029.7 ***	147	7.0	0.91	0.89	0.10	1115.7
Two-factor model (top, mid and PRESOR together)	3324.7 ***	149	22.3	0.68	0.63	0.19	3406.7
One-factor model (all variables together)	4269.8 ***	150	28.4	0.58	0.52	0.22	4349.8

*** *p* < 0.001.

**Table 3:** Descriptive Statistics Results and Correlation Matrix (N = 561).

Variables	Mean	S.D.	1	2	3	4	5	6	7	8
1.Gender	1.56	0.49								
2.Age	1.45	0.49	0.12							
3.Tenure	2.23	1.22	−0.05	0.12 **						
4.Education	2.29	0.62	−0.08	0.07	0.57 **					
5.Top-level Responsible leadership	3.52	0.99	0.00	0.04	0.01	−0.08				
6.Mid-level Responsible leadership	3.36	0.97	0.00	0.01	0.00	−0.09 *	0.77(0.59) **			
7.Employee Pro-environmental behavior	4.09	0.70	0.02	0.04	−0.02	−0.07	0.48(0.23) **	0.49(0.24) **		
8.Perceived role of ethics and social responsibility	3.95	0.88	0.03	0.06	−0.04	−0.08	0.42(0.17) **	0.50(0.25) **	0.56(0.31) **	-

SD, standard deviation. ** *p* < 0.01, * *p* < 0.05, for each pair of variable the shared variance appear in brackets.

**Table 4:** Results for Hypothesis 1 and 2.

	Coeff.	SE	*t*	95% CI	
Control variables				LL	UL
Gender	0.01	0.06	0.33	−0.07	0.11
Age	0.04	0.06	0.93	−0.04	0.15
Tenure	0.02	0.03	0.56	−0.03	0.06
Education	−0.09	0.05	−1.79	−0.20	−0.00
Mediating effect					
Total effect	0.34	0.02	13.14	0.30	0.38
Direct effect (TOP → Pro-environmental behavior-Hypothesis 1)	0.18	0.04	4.51	0.11	0.24
Indirect effect (TOP → MID → Pro-environmental behavior-Hypothesis 2)	0.16	0.03	-	0.10	0.21

CI = confidence interval; LL = lower limit; UL = upper limit.

**Table 5:** Results for Hypothesis 4,5.

	Coeff.	SE	*t*	95% CI	
Conditional effects of TOP on pro-environmental behavior (Hypothesis 4)					
Low perceived responsibility (−1 SD)	0.32	0.05	6.35	0.24	0.41
High perceived responsibility (+1 SD)	−0.00	0.04	−0.08	−0.08	0.07
Conditional indirect effects of TOP on pro-environmental behavior (Hypothesis 5)					
Low perceived responsibility (−1 SD)	0.01	0.05	-	−0.10	0.12
High perceived responsibility (+1 SD)	0.17	0.05	-	0.05	0.20

CI = confidence interval; LL = lower limit; UL = upper limit.

## Data Availability

Not applicable.

## Appendix A

**Table A1:** Measurement model results.

Constructs and Items	Standardized Factor Loadings
Top-level responsible leadership (Top-level RL) Rhô = 0.88, CR = 0.89, AVE = 0.67 and α = 0.9	
The enterprise’s manager demonstrates awareness of the relevant stakeholder claims.	0.789
The enterprise’s manager involves the affected stakeholders in the decision-making process.	0.786
The enterprise’s manager weighs different stakeholder claims before making a decision.	0.865
The enterprise’s manager tries to achieve a consensus among the affected stakeholders.	0.847
Mid-level responsible leadership (Mid-level RL) Rhô = 0.92, CR = 0.92, AVE = 0.71 and α = 0.95	
My direct supervisor demonstrates awareness of the relevant stakeholder claims.	0.835
My direct supervisor involves the affected stakeholders in the decision-making process.	0.897
My direct supervisor weighs different stakeholder claims before making a decision.	0.881
My direct supervisor tries to achieve a consensus among the affected stakeholders.	0.852
My direct supervisor considers the consequences of decisions for the affected stakeholders.	0.786
Employee pro-environmental behavior (E-PEB) Rhô = 0.85, CR = 0.86, AVE = 0.55 and α = 0.87	
I print double sided whenever possible.	0.773
I bring reusable eating utensils to work.	0.796
I turn lights off when not in use.	0.588
I take part in environmentally friendly programs.	0.722
I put compostable items in the compost bin.	0.826
Perceived role of ethics and social responsibility (PRESOR) Rhô = 0.90, CR = 0.90, AVE = 0.66 and α = 0.92	
Business ethics and social responsibility are critical to the survival of a business enterprise.	0.616
Business has a social responsibility beyond making a profit.	0.971
The ethics and social responsibility of a firm is essential to its long-term profitability.	0.945
The overall effectiveness of a business can be determined to a great extent by the degree to which it is ethical and socially responsible.	0.821
Social responsibility and profitability can be compatible.	
